# Provincial Hospital Arrangements

**Published:** 1914-08-22

**Authors:** 


*(From our Special Correspondent.)*

*(From our Special Correspondent.)*

### Provincial Hospital Arrangements.

### BARNSLEY.

*(From our Special Correspondent.)*

Already the local response to the Prince of Wales's appeal is worthy of the traditions of the town, and, amongst other contributions, £1,000 has been sent by the members of the Barnsley Co-operative Society. The local Patriotic Fund for the immediate relief of distress, particularly amongst the families of Territorials and Reservists, already amounts to over £1,500.

In reply to an inquiry from military headquarters at York, the Beckett Hospital are prepared to provide sixty beds for any wounded that may be sent for treatment. The Town Clerk also informs us that the Corporation are prepared to find forty-five beds at Lunwood Isolation Hospital, and the Board of Guardians another 100 at the Union Infirmary. Mrs. J. M. Spencer Stanhope, of Cannon Hall, Barnsley, is taking a very active part in organising the formation of a Voluntary Aid Detachment in connection with the West Riding of Yorkshire section of the Territorial Branch of the St. John Ambulance Association.

The Earl of Crawford has placed Haigh Hall at the disposal of the War Office as a convalescent home and a house in Wigan as a hospital. He has undertaken to defray the cost of upkeep.

### BATH.

*(From our Special Correspondent.)*

Acting upon the resolution of a meeting of the district vice-presidents held at Taunton, "that the Voluntary Aid Detachments of Bath should hold themselves in readiness to equip a temporary hospital if necessary," the Bath branch of the British Red Cross Society has rapidly put its organisation to work, and, co-operating with the local St. John Ambulance Association, which has placed two Voluntary Aid Detachments at its disposal, detailed arrangements have now been made to cope with any likely emergency.

The Royal Mineral Water Hospital, which is centrally and conveniently placed in the Upper Borough Walls and has accommodation for 150 beds, has been offered to the War Office, which, on acceptance, will provide the first hospital. Two houses have also been offered as hospitals, one at Lansdown by Mrs. Huth, which would provide twenty-five beds; the other, offered by Mr. Matthews at Westfield, for twenty beds; while should there be need for more beds the committee hope shortly to have the offer of the use of the mansion at Prior Park, a building—now in disuse—erected by the renowned Ralph Allen in order to display the virtues and value of Bath stone. When it is stated that the building has a frontage of some 1,300 feet it will be seen that the accommodation would be ample for any call that might be made upon the society's resources. The nurses enrolled are divided into two distinct divisions, the one those who are "trained," a definition which the committee interpret as applying to those who have received at least twelve months' training in a hospital of not less than fifty beds. These will take up ward nursing. The other division comprises those who, having no knowledge of nursing, are, however, desirous of becoming trained as Red Cross nurses. For these there are classes in first aid and home nursing taken by the local medical men, and the nurses after this instruction and the obtaining of certificates will become members of the society, to be used as occasion shall demand. There are also emergency classes in nursing for those willing to assist the ward nurses in bed-making, washing of patients, etc., and a number of ward maids, scrubbers, and cooks are also enrolled. Another valuable training class is for those who, not being able to become Red Cross nurses, are willing to be trained in order to be able to as far as possible take up for the time being the work of the district nurses, and so relieve them for service at the front and for Red Cross work in the hospitals.

With regard to the equipment of these emergency hospitals the names have been enrolled of persons who, when called upon, will be willing to give bedsteads, bedding, linen, etc., as well as money for the purchase of the necessary hospital furniture. An offer has been accepted from Miss Bickerton, a qualified chiropodist, to whom all nurses, whether working in the Red Cross hospitals or proceeding to the front, will be enabled to go during certain hours each day for advice and, if necessary, treatment.

In the men's work in connection with the Society there are, broadly speaking, two sections—the one to augment the St. John Ambulance Detachment in the district—which has already sent some of its members to the front, and is expecting further calls—by providing additional stretcher-bearers, who are being trained by the Assistant Commission of the St. John Ambulance Association for transport work, which will include the lifting of wounded, the loading and unloading of wagons, and the removal of wounded from trains; in enrolling these additional men a condition has been imposed that none will be accepted who are unable to mobilise at short notice. All enrolling are asked to turn out, if called upon in emergency, in two hours.

The second men's division is that known as the Men's Miscellaneous Section, which comprises those who are willing to take up any particular piece of work for which they individually are best qualified. These consist of clerks, porters, carpenters, pharmacists, etc., and the services of motor-cyclists have been secured, while the local scouts are acting as messengers.

The work of the Society in providing garments and requisites for the soldiers and sailors is being carried on by establishing working guild centres in various parts of the district; a sample-room is provided at the Guildhall, where materials may be seen and, if desired, purchased direct; all garments are to be made to a standard pattern, and for those who desire it, and purchase their materials from the sample-room, the shirts will be cut out by machinery. The Mayoress has, in addition, a fund for the purchasing of materials for those who, while unable to provide them themselves, are willing to work. There is also an excellent organisation for girls who are unemployed, or who may become unemployed, on account of the war, work being given to them in making the garments which may be then purchased by those desirous of helping, but who have no time for working up materials.

Miss Jackson is the local hon. secretary of the Red Cross Society, and Miss Hill, who is an Army nurse, and served for two and a-half years in the Boer War, is acting as lady superintendent, in the absence of Lady de Blaquiere, the Vice-President of the Bath and District branch of the Society. An excellent lead and invaluable advice is being given by the medical Mayor, Dr. Preston King.

### BARNSTAPLE.

*(From our Special Correspondent.)*

The committee of the North Devon Infirmary, Barnstaple, have offered an empty ward of fifteen beds to the War Office and Admiralty for use. The "Miller Institute," which is a good building standing in its own grounds, has been offered as a hospital. It will be prepared at short notice. It will hold thirty-nine patients. There is a Voluntary Aid Detachment in Barnstaple of about forty members. There is also a Voluntary Aid Detachment at Braunton, a village five miles out, and this detachment totals twenty. They keep two lofts always as practice wards, one as first-aid and lecture room, the other as ward with beds and lay figures. Both these detachments have many capable women. The latter has made arrangements to have a school when required.

### A SOUTHERN TERRITORIAL HOSPITAL.

*(From our Brighton Correspondent.)*

The 2nd General Hospital (R.A.M.C.T.), one of the twenty-three Territorial hospitals in the United Kingdom, has been mobilised at Brighton. The order for mobilisation was received from the War Office on Tuesday, August 4, by the Administrator, and he and his staff, consisting of a lieutenant and a quartermaster, immediately got to work to arrange all details.

Firstly, as to staff. Eighteen doctors, consisting of two lieutenant-colonels, four majors, and twelve captains, were called up. They were mainly members of the medical, surgical, or specialist staffs of the hospitals in the town, and were all ready and eager to help. The N.C.O.'s and men, in peace-time forty-three, were rapidly recruited up to war strength—110—and supplied with uniform. No difficulty was found in obtaining the requisite number. The nursing staff, consisting of a principal matron, a matron, thirty sisters, and about ninety nurses, are drawn from all parts of the county, including the local hospitals. Not all have been sent for yet, but quite a sufficient number for the present work have arrived. They are all lodged at a convent situated at a convenient distance from the hospital.

The hospital building itself is the New Grammar School, consisting of two blocks, and placed in a healthy, open part of Brighton. The wards are well arranged, there are rooms for the various officials, medical and nursing, and an operating theatre, suitable for all ordinary requirements, has been equipped. When it is complete there will be 520 beds, but not all of these are ready at present. Within a week of mobilisation the authorities were in a position to take in 260 patients. Not all the 520 beds can be placed in one building, and a Council school close by has been requisitioned. This is at present partly used as a barracks for the men to sleep in, and the remainder is rapidly being transformed into a section of the hospital.

### BRISTOL.

*(From our Special Correspondent.)*

We learn that the following members of the Bristol Infirmary staff have volunteered for service:—Lieut. R. S. Stratham, M.D., 3rd Field Ambulance, Aldershot; Lieut. H. W. Goodden, M.D., 4th Stationary Hospital, Woolwich; Lieut. C. C. Harrison, Lieut. C. M. Campbell, Lieut. H. J. Orr-Ewing, Lieut. C. B. Hogg, 5th General Hospital, Portsmouth.

*Territorial Forces.*—Lieut.-Col. J. Paul Bush, C.M.G., M.S., officer commanding 2nd Midland General Hospital; Lieut. C. F. Nichols, 2nd South Midland General Hospital; Capt. R. C. Clarke, M.D., Medical Officer of 4th Gloucester Regiment; Lieut. C. E. K. Herepath, M.D.; Lieut. J. P. I. Hardy, F.R.C.S., 3rd South Midland Field Ambulance; Lieut. R. A. Kerr, M.B., Medical Officer Royal Engineers.

A Voluntary Aid Detachment has been formed of the members of Bristol University, and the services of Dr. Baker and Mrs. de Soyres have been secured for training the members in first aid and nursing. At a recent meeting Lady Owen said that probably the convalescents would be drafted from Plymouth to Bristol, and their idea was to place a nurse, assisted by a probationer, in charge of seven or eight patients in the various houses offered for convalescent homes.

### CAMBRIDGE.

*(From our Special Correspondent.)*

Not only, as we noted last week, is Cambridge the centre at which the chief hospital and depôt of the Voluntary Aid Detachments is placed; but in addition the services of Addenbrooke's Hospital, Cambridge, have been offered to the War Office authorities during the present national crisis. The principal matron, Miss C. Crookenden, matron of Addenbrooke's, the matron, Miss Newton, sisters and nurses of the 1st Eastern General Hospital Territorial Force Nursing Service, in number about 100, have been mobilised, and have their quarters at Downing College.

In addition to the above the whole of the honorary physicians and honorary surgeons of Addenbrooke's have been mobilised, and are holding themselves in readiness for service. Among those mobilised for service are the following members of the hospital staff: Mr. H. F. Rutherford, in-patient and stores clerk; Mr. C. Charter, senior dispensary apprentice; and Mr. F. Duke, clinical laboratory attendant.

Addenbrooke's Hospital has for the past five years undertaken year by year to supply six nurses to supplement Queen Alexandra's Imperial Military Nursing Service in the event of war or national emergency, and these nurses have been requested to hold themselves in readiness for orders, and two have since been definitely called upon for service. In addition Addenbrooke's Hospital has for some years past undertaken to supply two nurses to supplement Queen Alexandra's Royal Naval Nursing Service in the event of war or national emergency.

In case of great necessity it is expected that hospital accommodation in connection with the Territorial R.A.M.C. will be provided at one or more of the principal colleges, in addition to the service offered by Addenbrooke's Hospital.

The Red Cross Society is very active in making preparations for supplying a number of beds in various centres, augmenting the nursing service, and by providing a supply of all the necessary garments required for the sick and wounded.

There is universally throughout the town and country a great effort being made to provide an adequate supply of garments, etc., required by the sick and wounded amongst our soldiers and sailors who may be brought to this district and elsewhere.

### ESSEX.

*(From our Special Correspondent.)*

The relatively large grounds, which amount to some two acres, surrounding the Essex County Hospital at Colchester have led the committee not only to offer as many beds as possible to the Admiralty and the War Office, but also to suggest that the grounds themselves might be tented over if required. Owing to the close proximity of Harwich the hospital Secretary, Mr. Alfred G. Buck, expects that in due course every available bed may be required by the Government. Colonel Colvin, C.B., county director of the Essex Branch of the British Red Cross Society, expressed the view last week that Harwich would be an extremely important place. [In connection with the grounds it may be suggested that they form a very useful opportunity for weighing the scheme of open-air hospital treatment, in the shape of semi-open shelters, on the lines laid down the other day by Dr. Saundby, of Birmingham, and summarised in our Notes this week.]

The Essex Branch of the British Red Cross Society consists of seventy-three Voluntary Aid Detachments with a *personnel* of about 2,000, of whom almost three-fourths are women. Three of these detachments are formed from St. John Ambulance Brigade. The county is divided into divisions corresponding generally with the police divisions. Colonel R. B. Colvin, C.B., is the county director. Each division is controlled by a vice-president and assistant county director. The offices of the branch are now at Chelmsford, and the Honorary Secretary is Colonel Coleman, V.D.

Since the commencement of the war many offers of private houses, institutions, and other buildings for use either as hospitals or convalescent homes have been made, and already accommodation could be found for 2,000 patients in the county, and offers continue to come in.

At Harwich the Great Eastern Hotel has been requisitioned as a hospital; 120 beds have been prepared. The local detachments (men), under Mr. T. W. Etherden, late of the Essex Yeomanry, and women, under Mrs. N. Brooks, are already employed. At Brentwood during the concentration of the East Anglian Division a temporary hospital containing twenty beds has been formed at the Grammar School. Several cases, some of a serious nature, have been attended to. A depot will be shortly formed at Chelmsford where voluntary contributions in the shape of stores and clothes will be collected.

### HOSPITAL CO-OPERATION IN EXETER.

(*From our Special Correspondent.*)

A good instance of hospital co-operation is seen in Exeter, where the committee of the Royal Devon and Exeter Hospital have acceded to a request from the Royal West of England Eye Infirmary in Exeter to have their patients in the event of that institution being required to place its beds at the disposal of the Voluntary Aid Detachment. Nine members of the nursing staff have left for active service, and innumerable offers of houses for use as hospitals and convalescent homes in Devonshire have been received by the Devonshire branch of the British Red Cross Society.

### GRAVESEND.

(*From our Special Correspondent.*)

Immediately war was declared the beds at the Gravesend Hospital were placed at the disposal of the Naval and Military authorities at Chatham. This necessitated prompt action in augmenting the number of beds at the hospital—almost doubling the ordinary number—so that the civilian work should not suffer in any way, it being contended, and very rightly so, that it would be an inopportune time to decrease any facilities for treating the sick poor. Most assuredly the tendency should be the other way. Offers of single bedsteads, bedding and linen were invited, and very quickly nearly the whole of these requirements were furnished from voluntary sources. Numerous and varied were the offers of help, and it was extremely difficult to deal with all of them, but the right spirit was there, and names and offers were duly registered, to be utilised later if necessary. One old gentleman, unable to think of any other way, asked for his name to be put down as a night watchman on delirious patients! Several persons offered houses for convalescents; and one lady, an old hospital nurse, writing from a village near by, asked to have "two chronic or convalescent patients, and so relieve your beds." Notwithstanding nurses and others being called to the Colours, the gaps were quickly filled, several trained nurses applying as volunteers. In addition, a band of sixteen ladies, drawn from the many who have offered their services, are working hard taking in as much as possible—seeing practical work—in the hope that in an emergency their services may be requisitioned, and that they may make their presence felt as first-year probationers. The needle, too, has not been idle, for some of the ladies of the ladies' association attached to the hospital have worked incessantly getting linen, shirts, pyjamas, and such necessary articles ready.

The local Voluntary Aid Detachments have done splendidly, and have turned the technical school into a hospital of 200 beds. This, with every single part of its equipment from voluntary sources, is a task of no small magnitude. Indeed there must be hard workers in the Voluntary Aid Detachments, for according to the local paper the Mayor said, at a meeting called to centralise all efforts, that he believed their aim was to equip 1,000 beds in the borough.

### GUILDFORD.

(*From our Special Correspondent.*)

The Red Cross Society and the Hon. Medical Staff have formed a committee to equip a large new County School, opposite the Royal Surrey County Hospital, in which to carry on the work of nursing the wounded, under the immediate control and management of this hospital. The operating theatre and *x*-ray department would be used, and the whole of the supplies, etc., would come from the institution. A careful account of the quantities and cost would be kept.

There is reason to believe that the Army authorities intend to make Guildford an important centre for this work, and inquiries are in progress with a view of being able to arrange for a much larger number of beds than the 100 proposed, if required.

Mr. F. F. Smallpiece, chairman of the Royal Surrey County Hospital Management Committee, presided over the recent meeting. The work will only be started upon a request from the War Office. Dr. Mitchell, in giving the views of the hon. medical staff of the County Hospital, considered that, if run in connection with the County Hospital, the *x*-ray room, operating theatre, and dispensary could all be utilised. The County

Hospital would be short of house surgeons, but the hon. medical staff would undertake to perform the duties in their absence. The school should be treated as an annexe of the hospital. Some of the trained nursing staff could then act as ward sisters, with members of the Voluntary Aid Detachments working under them.

A joint committee of the County Hospital Management Committee, of the Hon. Medical Staff, and of the Red Cross Society was formed.

### IPSWICH.

*(From our Special Correspondent.)*

The evils of overlapping in the provision of hospitals for, and the subsequent care of, the wounded were discussed at a meeting of the medical men of Ipswich, held at the East Suffolk and Ipswich Hospital last week. This meeting, called by members of the honorary medical staff of the hospital, was largely attended by the practitioners in the town, and the conveners had had the wisdom to invite representatives of the Army and Navy medical departments, whose views at such a critical moment were of the utmost value.

The multiplicity of small units for early treatment of the wounded was deprecated as tending to an overlapping of efforts and an undoubted accentuation of the difficulties of adequate medical attention, although all agreed that these small units would be valuable for the treatment of convalescents.

There already exists a definite arrangement by which the East Suffolk and Ipswich Hospital will place some 100 beds at the disposal of the wounded who will be sent in by the naval authorities at Shotley. The general practitioners were advised to await directions of the War Office before making any definite arrangements for wounded soldiers.

It is evident to all concerned that the great factor in the whole question is the necessity for a trained nursing staff, and it appears that the general hospitals are at present the only institutions where the number of trained nurses is adequate compared with the number of beds provided.

### LANCASHIRE.

*(From our Special Correspondent.)*

The District Infirmary and Children's Hospital, Ashton-under-Lyne, in which is situated the depôt of the Manchester Regiment, has resolved to place its resources at the disposal of the War Office as far as its limited accommodation will allow.

The Board of Governors also decided to admit local ambulance and Red Cross nurses to the District Infirmary at stated times, and to provide facilities for training them in surgical work.

Mr. H. G. Hall has offered the use of Thornfield Hall for the treatment of sick and wounded soldiers. Thornfield Hall is close to the Infirmary. The Ashton-under-Lyne Division of the St. John Voluntary Aid Detachment have been requested to establish a temporary hospital in Ashton. The matter is in the hands of the commandant, Dr. Corns. Arrangements have already been made for using the Mechanics' Institute as a temporary hospital. There will be accommodation for twelve beds, and Dr. Corns, the honorary physician to the Nursing Association of the St. John Ambulance Brigade, Ashton Division, will be in charge.

Dr. Talbot has placed his residence, Early Bank, at the disposal of the War Office for a hospital for sick or wounded soldiers. This hospital would accommodate thirty beds, and Dr. Talbot states that practically the whole of the medical men in the town would give their assistance.

### LEICESTER.

*(From our Special Correspondent.)*

Leicester forms one of a group of twenty-three towns selected for the formation of base hospitals under the Territorial scheme, and is the headquarters of the 5th Northern General Hospital. In its turn the hospital is one of a group of similar hospitals in the Northern Command, of which Col. Astley V. Clarke, one of the physicians of the Leicester Royal Infirmary, is the Director, with headquarters at Derby. In Leicester the building formerly used as the County Asylum, but now for several years unoccupied, and which stands on an eminence adjoining the Victoria Park, has been placed at the disposal of the military authorities by the county authorities as a base hospital. The Commandant is Major L. K. Harrison, and the medical officers are largely drawn from the staff of the Leicester Royal Infirmary. Dr. Henry fills the responsible post of registrar, Mr. S. P. Pick is the architect, Miss C. E. Vincent, matron of the Leicester Royal Infirmary, principal matron, and Miss Hannath, of the Wolverhampton General Infirmary, matron. Provision will be made for 520 patients and about 100 sisters and nurses. An enthusiastic Ladies' Committee has been formed by Mrs. L. K. Harrison, and a Games and Entertainment Committee has been organised. The necessary alterations to the hospital will take time to complete, and in the interim the Board of Governors of the Leicester Royal Infirmary have arranged to provide at short notice up to 100 beds in case of emergency, and to undertake the sterilisation of all dressings, etc., for the base hospital. Arrangements for the new hospital are being rapidly pushed on, and tenders have been called for for the supply of provisions, etc. At the half-yearly meeting of the Leicester and County Saturday Hospital Society on Saturday, the Convalescent Homes of the Society were readily placed at the disposal of the hospital authorities for convalescent sick and wounded soldiers. A similar offer has also been made by the authorities of the Charnwood Forest Convalescent Homes. The St. John Ambulance Society, the Red Cross, and other Societies are rendering useful service in providing for the instruction of many voluntary workers in the care of the sick and wounded and the principles of elementary nursing.

### LINCOLNSHIRE.

*(From our Special Correspondent.)*

On the outbreak of war the 4th Northern General Hospital (Territorial) was mobilised. Lieut.-

Colonel W. H. B. Brook, R.A.M.C., the administrator, immediately commenced work. The Grammar School was taken over and structural alterations, as well as the erection of twenty-one temporary huts, each to have accommodation for twenty patients, were at once commenced. When finished this hospital will be able to deal with 520 patients, and could at once admit 400. The staff consists of twenty-one medical men in the Territorial Service drawn from the practitioners in the town and ninety-one Territorial nurses, who will all be mobilised. Fortunately the new nurses' home in connection with the County Hospital was almost finished, and accommodation for sixty nurses has there been provided, the rest being housed in neighbouring buildings.

### MIDDLESBROUGH.

*(From our Special Correspondent.)*

Being near the North-East coast we are almost certain to have a number of wounded sent to this district. Mr. E. C. Smith, Secretary of the North Ormesby Hospital, Middlesbrough, has had a letter from the Admiralty asking him to place a certain number of beds at their disposal, and requesting that these beds shall be kept ready for immediate occupation without further notice. The other local hospital, the North Riding Infirmary, has also volunteered to place a certain number of beds at the disposal of the Admiralty; the workhouse infirmary has done the same; the Cleveland Asylum has offered its small hospital; and Messrs. Bolckow, Vaughan & Co., Ltd., who are really the owners of the Eston Hospital, have made a similar offer. In addition a large private residence belonging to Bolckow, Vaughan & Co., at Redcar, a seaside resort about ten miles from Middlesbrough, has also been offered to the Admiralty for either wounded soldiers or convalescents; the Coatham Convalescent Home governors have also stated that they will be pleased to discharge all their convalescents and place the home, containing 180 beds, at the disposal of the Admiralty.

A similar offer has also been made by the local authorities of the floating hospital on the Tees. In addition to these, the town authorities have made preparations for converting certain schools, etc., into hospitals should they be required. The latter arrangements are being carried out by the local members of the Red Cross and the St. John Ambulance Associations.

### OLDHAM.

*(From our Special Correspondent.)*

The offer of the governors of the Oldham Royal Infirmary of fifty beds, with housing, nursing, and medical attendance during the present war, has been cordially accepted by the Secretary for War. In addition to this, four sisters (two of whom have already gone) and several nurses are awaiting instructions to proceed to various destinations. The Oldham Royal Infirmary has in daily attendance about thirty Red Cross nurses, who are receiving instructions in the method of treating the sick and injured, so as to be in readiness for all contingencies that may arise.

### PORTSMOUTH.

*(From our Special Correspondent.)*

Many members of the honorary medical and surgical staff of the Royal Portsmouth Hospital have been summoned or volunteered for service at the Military and Territorial hospitals, but arrangements are being made for the treatment of all "cases" of a serious or urgent nature. Four sisters, one staff nurse, two private nurses, one dispenser, and the laundry engineer have been called up for service. The wards are fully occupied, and every effort will be made to maintain the efficient working of the available beds.

The committee have already rendered assistance to the military authorities by admitting patients from the Military Families' Hospital, which is being prepared for the reception of officers. The preparations at Portsmouth district for the reception and treatment of the sick and wounded, in addition to the permanent Military and Naval hospitals at Cosham, Netley, and Haslar, include the 5th Southern General Hospital's Section of the Territorial Force, established at the Secondary Schools, Southsea. Five hundred beds will be provided, and the nursing arrangements are in charge of the principal matron, Miss Alcock, of the Royal Portsmouth Hospital, who has over ninety nurses now available.

### HARTSHILL, STAFFORDSHIRE.

*(From our Special Correspondent.)*

At the North Staffordshire Infirmary two wards of twenty-two beds each have been offered to the Government for the use of the wounded, and for a temporary period arrangements have been made for practical instruction to be given in the casualty department and in the wards for a few hours each day for a period of one week to Red Cross nurses who hold both First Aid and Nursing certificates. Two house surgeons have volunteered, and also some members of the voluntary staff, in accordance with the arrangements made for certain members of the staff, who desired it, to offer their services. The matron of the hospital, Miss MacMaster, and the sister from the accident ward are at Harwich, having been specially summoned to organise the emergency military hospital there. There is a fairly sufficient supply of drugs and other necessaries, but if the price of these remains high, expense and difficulty may arise in replenishing it.

The following may be useful to some of our readers as showing the arrangements made for the attendance of Red Cross nurses at the North Staffordshire Infirmary, as typical of the hospital requirements in this respect:—

### Regulations for the Attendance of Red Cross Nurses.

1. For a temporary period, the duration of which has not yet been fixed, arrangements have been made at the North Staffordshire Infirmary for practical instruction to be given as under to Red Cross nurses who hold both the First Aid and Nursing certificates.

2. Six Red Cross nurses will be admitted for attendance in the surgery for three days at the beginning of the week, and for instruction in wards 1, 2, or 7 for the three remaining days of the week, or *vice versa.*

3. Ordinary Red Cross uniform with sleeves must be worn.

4. Red Cross nurses attending at the North Staffordshire Infirmary become for the time probationary members of the nursing staff of that hospital and are subject to all the rules and regulations for nurses as far as they relate to their work.

It must be understood that Red Cross nurses attending must in all cases implicitly obey the instructions and directions of the sister in charge.

5. Nurses attending at the surgery must arrive punctually at 9 A.M. and leave punctually at 11 A.M.

Nurses attending in the wards must arrive at the ward arranged for them promptly at 5 P.M. and leave promptly at 7 P.M.

6. Unpunctuality or failure in other ways to carry out the above regulations will render the nurse liable to be immediately suspended from attendance at the hospital.

7. Attendance cards must be brought each day and shown to the sister in charge of the particular department or ward at which the Red Cross nurse is due.

### STALYBRIDGE.

*(From our Special Correspondent.)*

THE Great Central Wagon Works, Dukinfield Hall, are understood to be building two hospital trains to the order of the War Office.

Seven members of the Stalybridge and Dukinfield Division of the St. John Ambulance Brigade have been passed for special service with the R.A.M.C. for twelve months, or for such period as the war shall last. Mr. Harry G. Hall has offered Kelsall House, Stalybridge, as a relief hospital.

### TORQUAY.

*(From our Special Correspondent.)*

THE board of management of the Torbay Hospital has offered the Admiralty fifty beds. The number is to be increased should pressing emergency arise. The offer has been accepted.

The committee of the Rosehill Children's Hospital has offered to take all children from the Torbay Hospital, thus enabling the large children's ward to be fitted and used as a surgical ward for men. Arrangements have also been made for the nurses to sleep out in order that their rooms might be used as single-bed wards for officers should the emergency arise. By re-arrangement the number of beds can be increased to 100. Surgeon-Captain S. J. A. H. Walshe, house surgeon to the Torbay Hospital, has left for Aldershot. Three of the nurses have also gone. The Torquay branch of the Red Cross Society has fitted up the large assembly hall at the new Town Hall as a hospital to accommodate fifty patients. The Town Hall almost adjoins the Torbay Hospital. There are in the hospital ordinarily sixty-four beds for the use of patients, and the average number occupied is thirty-seven. Altogether it is calculated that 125 beds could be provided. Allowing thirty-seven—the average number—for ordinary patients, there would be eighty-eight for use by soldiers and sailors, and the number could be increased to 100.

The committee of the Western Hospital for Consumption, Torquay, has offered fifty beds to the War Office. The War Office has written for Torquay to prepare a convalescent home at once, and Rockwood, the residence of the late Mrs. N. B. Stewart, will be adapted for this purpose.

The assembly room at the new Town Hall, with the consent of the Corporation, has been transformed into a divisional hospital, which has been equipped by the Red Cross Society. Mrs. Acton is the lady commandant, and the medical staff consists of Dr. and Mrs. Payne, Dr. Wightwick, Dr. Lacey, Dr. Winter, Dr. Scott Gillett, and Dr. Hugh Steele. The quartermasters are Captain Chandler, Miss Honeywill, Miss Phillpotts, and Miss Collins. Miss Keary is the hospital housekeeper, and the cooking arrangements are under the personal superintendence of Mrs. Dutton. Dr. Dunlop has charge of the transport. Sergeant-Major Tomney has been appointed ward master, and the staff includes six trained nurses and hospital orderlies. In accordance with instructions the hospital was ready at midday on Monday 10th inst. The services of the whole of the members of the three local Voluntary Aid Detachments of the British Red Cross Society—Devon 6th, 22nd, and 26th—will be available as required. The hospital ward contains fifty beds in four rows. There are in other parts of the Municipal buildings dining and sitting rooms, with sleeping accommodation, for the nurses. The kitchen has been fitted with several gas stoves for cooking purposes.

The local division of the Ambulance Brigade, of which Dr. Dunlop is the superintendent, is to undertake service. On receipt of a message from Dr. Grimbley, the Deputy Commissioner, asking for the names of members of the brigade who were willing to enlist for one year in the Royal Army Medical Corps, to serve either at home or in a special expeditionary force abroad, the members were immediately called together. In response to the Chief Commissioner's appeal, no fewer than seventeen out of a total membership of thirty-two volunteered for active service.

### TUNBRIDGE WELLS.

*(From our Special Correspondent.)*

AT the General Hospital the committee have made arrangements for the accommodation of fifty sick or wounded troops. This offer has been communicated to the military authorities of the division, who will accept the beds should necessity arise. To be able to offer this number the committee will be compelled to add to their number of beds by the equipment of the out-patients' waiting-hall, a large and lofty building, the beds, mattresses, and pillows for this extra number being provided gratis by a local tradesman, Mr. E. Oliver.

Dr. C. Vise, Major R.A.M.C., T.F., has been with the troops since the declaration of war. He is senior physician at the General Hospital. Dr.

R. M. Ranking, retired Captain R.A.M.C., has been recalled to service with the Regular Army.

The Eye and Ear Hospital has offered ten beds for the use of the troops. Mr. Beale and Mr. Clapham have placed houses in large gardens at the disposal of the Red Cross Society for hospital purposes. Bidborough Court, a large house some three miles from Tunbridge Wells, is also being equipped as a nursing centre. Lady Margaret Cecil has very kindly offered to receive patients from the General Hospital, who can be looked after satisfactorily at her house at Burwash, and thus relieve the beds for urgent cases.

At Tonbridge the Voluntary Aid Detachment are making complete arrangements for the nursing of the sick and wounded. Fifty beds will be at their disposal at the school sanatorium, and if further accommodation is needed the school house and museum will be available. Five of the sisters and one temporary sister at the General Hospital are attached to the Territorial Nursing Service, three have taken up their duties in that capacity, and the others have orders to be in readiness. Their places on the nursing staff have been filled for the time being. To enable the local Red Cross nurses to have some ward experience, the committee of the General Hospital have allowed four at a time to do duty in the wards; after three days another four take their places, and so in time all will have some practical ward work.

### WAKEFIELD.

*(From our Special Correspondent.)*

Preparation has already been made at Clayton Hospital for two wards to be set aside with twenty beds in each, and the same are already furnished and equipped, and are ready at a moment's notice for the receipt of wounded or convalescent soldiers or sailors. Members of the St. John's Voluntary Nursing Association are attending daily at the Clayton Hospital and receiving lectures from the hon. physicians, and assist in the wards and out-patients' department.

The St. John's Nursing Association is very strong here, and has a large number of members, and they are not only obtaining practical experience by attending at the hospital daily, but are also receiving lectures from medical men at schools in the city, and are preparing to equip sixty beds, thereby fulfilling the request received from the War Office that 100 beds should be equipped at Wakefield altogether.

It is believed that some arrangements are about to be made at the Wakefield Workhouse Infirmary whereby a certain number of beds can be set aside.

### WALSALL (STAFFS).

*(From our Special Correspondent.)*

New Voluntary Aid Detachments are being formed in connection with the Walsall District Branch of the British Red Cross Society, and the executive committee of the Walsall and District Hospital have placed at their disposal accommodation for at least thirty of the sick and wounded. Two wards which have been closed for some years owing to lack of support will provide this accommodation, and they are quite ready for use, so that the ordinary work of the hospital can be continued without interruption. Should the necessity arise it is probable that more beds will be offered for the same purpose. Offers of beds have also been received by the Red Cross Society from the Victoria Nursing Institution, Walsall, and Miss Barton's Private Nursing Home, Walsall, but the number of beds in each instance is not to hand.

Four members of the hon. medical staff, Drs. C. J. Caddick, J. G. Cooke, Footner, and Sydenham, have volunteered for service with the forces. The remaining members of the honorary staff have taken their place. The re-opening of the children's ward, which was made financially possible when the war began, will be postponed, and the ward will be used for the Red Cross purposes.

As to hospital supplies, the contracts came into operation on July 1 last, but Mr. F. J. Cotterell, the chairman, does not think that the terms can be enforced in the circumstances. Where contracts have been rescinded, as in two cases, the committee has instructed the secretary, Mr. Frank Inch, and the matron to purchase as best they can in the open market.

### YARMOUTH.

*(From our Special Correspondent.)*

Dr. Herbert Mayo, assistant surgeon to the Admiralty at Great Yarmouth, has been organising for the past fortnight, in connection with the local branch of the British Red Cross Society, arrangements for receiving sick or wounded soldiers and sailors. The Drill Hall was furnished last week with fifty beds, some of which have since been removed to other buildings. The Sailors' Home is ready to receive twenty patients, and St. Mary's (R.C.) Schools a similar number. Both institutions are in charge of ladies attached to the Voluntary Aid Detachments. The deputy Mayor, Mr. R. G. Westmacott, and Mr. Harold Chamberlin have been placed in charge of the organisation of transport. The commissariat arrangements are in charge of Mr. H. S. Bracey and Mr. G. R. Talbot. Although a large number of wounded are only expected to be landed in the event of an action taking place close at hand, when the majority would go to Chatham in hospital ships, the patrols of destroyers may, however, bring in a few sick and wounded. Fleet-Surgeon Miller, R.N., has prepared, and is in charge of a reception station at Fish Wharf. He will decide to which hospital each case shall be passed on.

The General Hospital at Yarmouth, of which Mr. Richard F. E. Ferrier is the honorary secretary, has fifty beds in readiness for major cases, and minor cases can be received at the Gorleston Cottage Hospital and at a local nursing home, although these two last have not been requisitioned as yet. Dr. Herbert Mayo is chairman of the local executive of the British Red Cross Society. Mr. S. L. Moore, who has charge of the loans and equipment of the local hospital, is registering the male assistants; 800 offers of help have been received. It is claimed locally that the Yarmouth Detachment of the Red Cross Society was the first county organisation to mobilise.

*(For want of space several Scotch and other reports are held over.)*

**[The Editor's Letter-Box will be found on page 3 of the "Institutional Worker."]**

